# Purinergic Signalling in Immune System Regulation in Health and Disease

**DOI:** 10.1155/2015/106863

**Published:** 2015-03-01

**Authors:** Jean Sévigny, Mireia Martín-Satué, Jesús Pintor

**Affiliations:** ^1^Département de Microbiologie-Infectiologie et d'Immunologie, Faculté de Médecine, Université Laval, Québec City, QC, Canada G1V 0A6; ^2^Centre de Recherche du CHU de Québec, QC, Canada G1V 4G2; ^3^Departament de Patologia i Terapèutica Experimental, Facultat de Medicina, Universitat de Barcelona, 08907 L'Hospitalet de Llobregat, Spain; ^4^Institut d'Investigació Biomèdica de Bellvitge, 08908 Barcelona, Spain; ^5^Department of Biochemistry and Molecular Biology, Faculty of Optics, Universidad Complutense de Madrid, 28037 Madrid, Spain

The concept of a purinergic signalling system was first proposed by Professor Geoffrey Burnstock over 30 years ago. This includes the cellular responses to purine nucleotides, such as ATP, and nucleosides, such as adenosine, that act as extracellular messengers playing a role through specific nucleotide and adenosine receptors in all systems. Indeed, in addition to their role in cellular metabolism, nucleotides as well as nucleosides are extracellular mediators that activate biological responses in all cells. Cells subjected to activation or shear or mechanical stress release nucleotides such as ATP, ADP, UTP, and UDP in large amounts. All cells can release nucleotides in a controlled fashion [1]. The mechanisms of nucleotide release have been the focus of intense research activities but are still not fully understood. While activated platelets and neurons release nucleotides by exocytosis, neutrophils, and T lymphocytes use pannexin-1 hemichannels for nucleotide efflux, some cells also constitutively release nucleotides.

Under normal conditions, blood levels of nucleotides are below 0.1 *μ*M, the level resulting from cell release, degradation by ectonucleotidases and reuptake of nucleotides after dephosphorylation by these enzymes [2]. There are many sources of extracellular nucleotides. In the circulatory system, red blood cells are a major source of nucleotides. The dense granules of the platelets contain a phenomenal amount of ATP and ADP approaching 1 M concentration that are secreted by exocytosis during activation. Nucleotides, primarily ATP, can be released by the endothelial cells and the smooth muscles cells of vessel walls. Nucleotide release can be triggered by shear stress, hypoxia and cell breakdown. As cytosolic ATP concentration is about 5 mM, rupture of the plasma membrane causes an increase in nucleotide concentrations near the injured cells. These mechanisms represent only a few examples of nucleotide release. Cells thus release large amounts of ATP, in a controlled manner but also when a cell is injured or dies which is particularly relevant in situation of inflammation and/or diseases.

Once released, nucleotides activate ubiquitously expressed ligand-gated ionotropic P2X receptors (P2X1–7) and G-protein-coupled metabotropic P2Y receptors (P2Y_1,2,4,6,11–14_) [1]. In addition, the cysteinyl-leukotriene receptor-1 and receptor-2 (CysLT_1_ and CysLT_2_) and GPR17 can also respond to nucleotides. P2 receptor subtypes differ with respect to selectivity towards nucleotides. All P2X receptors are activated by ATP, P2Y_2_ by ATP and UTP, P2Y_1_, P2Y_12_, and P2Y_13_ by ADP, P2Y_4_ by UTP, P2Y_6_ by UDP (in mouse also by UTP [3]), P2Y_11_ by ATP > ADP, and P2Y_14_ by UDP-glucose. In addition, adenosine, which results from the complete dephosphorylation of ATP and ADP by ectonucleotidases, activates P1-type receptors, which divide into four types: A_1_, A_2A_, A_2B_, and A_3_. These receptors are widely distributed and their activation has already been demonstrated to modulate a myriad of biological responses.

The responses induced by nucleotides can be modulated by cell surface enzymes generically called ectonucleotidases. Among them, the members of the ectonucleoside triphosphate diphosphohydrolase family (E-NTPDases) [4, 5] which convert tri- and diphosphonucleosides (ATP, ADP) to their monophosphate derivative (AMP). The latter is further hydrolysed to adenosine by ecto-5′-nucleotidase (CD73). Several other enzymes also modulate nucleotide concentrations, namely, nucleotide pyrophosphatases/diphosphodiesterases and alkaline and acid phosphatases. P2 receptor signalling can also be influenced by the ectokinases, that is, ectonucleotide diphosphokinases and adenylate kinases, that interconvert and regenerate nucleotides, respectively [2]. Hydrolysis thus switches off the nucleotide signal and plays a role in adenosine formation. These enzymes thus control relative concentrations of each nucleotide and nucleoside and, accordingly, regulate their effects.

Since its discovery, purinergic signalling has been shown to mediate a wide range of functions in health and disease, important among them being immunomodulation and inflammation. Recent advances have been made in therapies using nucleotide-related drugs in a broad range of pathological conditions such as acute and chronic inflammatory diseases. In this special issue, which is dedicated to purinergic signalling in immune system regulation in health and disease, we present 9 research papers and 4 reviews. The papers deal with the following topics.

By catalyzing the hydrolysis of extracellular AMP to adenosine, ecto-5′-nucleotidase/CD73 was shown to control vascular permeability and immune responses. By using double knockout mice lacking CD73 and another AMP-degrading enzyme, prostatic acid phosphatase (PAP), G. G. Yegutkin et al. show that PAP may have a synergistic role together with CD73 in the immune system by contributing to the lymphoid purine homeostasis and balance of leukocyte subpopulations in the lymph nodes and thymus. However, the absence of PAP alone did not have any significant effects outside the thymus.

S. D. S. Oliveira et al. show that schistosomiasis reduces peritoneal macrophage P2X7 receptor signaling which correlate with increased levels of TGF-*β*1 in infected mice as well as with reduced cell surface expression of P2X7. The involvement of P2 receptors in chemotactic responses of fibroblasts in the context of regulation of inflammatory responses and tissue damage repair is addressed by M. Pimentel-Santillana et al. in a paper that demonstrates that Prostaglandin E_2_ inhibits P2Y-dependent cell migration.

Data presented by L. Oliveira et al. with a rat model suggest that restoration of neuromuscular transmission and immune competence may be possible by targeting common adenosine deficits in patients with autoimmune myasthenia gravis. In another paper, C. Vieira at al. show that postinflammatory ileitis suppresses adenosine neuromodulatory control leading to acceleration of the gastrointestinal transit, which may last more than a week. In an attempt to identify biomarkers for endometriosis, an inflammatory estrogen-dependent complex disorder that is one of the principal causes of infertility in women, L. Texidó et al. demonstrate the presence of ectonucleotidase activities in the contents of endometriomas.

The contribution carried out by K. R. Higgins et al. indicates that the activation of a P2Y_2_ receptor present in a human monocytic cell line (THP-1) is very efficient inducing the chemokine CCL2. The effect carried out by the purinergic agonists is as good as the one triggered by lipopolysaccharide. Moreover, these researchers have found that polymorphisms of the P2Y_2_ receptors are important regarding the CCL2 secretion. In this sense, human macrophages expressing 312Ser-P2Y_2_ displayed significant UTP-induced CCL2 secretion above background compared to 312Arg-P2Y_2_ expressing macrophages.

Neuroinflammation in the central nervous system occurring during pathologies such as amyotrophic lateral sclerosis and multiple sclerosis is a severe problem associated with those pathologies. S. Amadio et al. studied the role of P2Y_12_ receptors in the neuroinflammatory condition, mainly on ramified microglia and myelinated fibers from primary organotypic cerebellar cultures or tissue slices from rat striatum, cerebellum, and spinal cord from symptomatic animals. These authors suggest that the modulation of P2Y_12_ expression might play a dual role as analytic marker of branched/surveillant microglia and demyelinating lesions and also that potentially could be a predictive value under neuroinflammatory conditions as those found in amyotrophic lateral sclerosis and multiple sclerosis.

Laser therapy, particularly low-level-laser therapy, is being used with success as a complementary treatment for inflammation and wound healing in the dermis. L. Wang et al. present a paper dealing with the effects of red-laser irradiation on extracellular ATP content of mast cells and dorsal root ganglia neurons. In this sense, the authors show that irradiation potentiates extracellular ATP presence in mast cells by promoting ATP synthesis and release, but on the contrary laser attenuates extracellular ATP amounts of dorsal root ganglia neurons by upregulating ecto-ATPase activity.

This issue also presents the following reviews. The review of F. Pedata et al. covers the role of the adenosine receptor A_2A_ in acute injury and neuroinflammation in brain ischemia. A. Guzman-Aranguez et al. review purinergic receptors in ocular inflammation pointing out to the use of purinergic agonists and antagonists as possible therapeutic targets for inflammatory eye disorders. In their review, P. J. Sáez et al. focus on the effects of different cytokines on the intercellular communication mediated by hemichannels and gap junction channels in antigen-presenting cells and their impact on purinergic signaling. Finally, A. R. Santiago et al. reviewed the role of the nucleoside adenosine in microglial proliferation, chemotaxis, and reactivity. The authors focused on the adenosine A_2A_ receptor and discuss its role in Parkinson's and Alzheimer's diseases as well as in glaucoma and diabetic retinopathy.

Although there is still much to learn about the precise role of the adenosine and P2 receptors in the immune system and in inflammation, there is now wider acceptance that adenosine and nucleotides have a real and important function in this regard. This timely collection of well-informed and insightful articles summarises where we are now and points the future directions we should take. As the purinergic field is growing rapidly and it is getting increasingly demanding to keep up with the literature, we hope that this special issue will not only be useful from this point of view but also will inspire new experiments that reveal further insights regarding adenosine and nucleotides in all the aspects related to inflammation and the immune system.
